# Oligodendrocyte-specific Argonaute profiling identifies microRNAs associated with experimental autoimmune encephalomyelitis

**DOI:** 10.1186/s12974-020-01964-5

**Published:** 2020-10-12

**Authors:** Qin Ma, Atsuko Matsunaga, Brenda Ho, Jorge R. Oksenberg, Alessandro Didonna

**Affiliations:** Weill Institute for Neurosciences, Department of Neurology, University of California San Francisco, 675 Nelson Rising Lane, San Francisco, CA 94158 USA

**Keywords:** MicroRNAs, Oligodendrocytes, Multiple sclerosis, Autoimmunity, Experimental autoimmune encephalomyelitis

## Abstract

**Background:**

MicroRNAs (miRNAs) belong to a class of evolutionary conserved, non-coding small RNAs with regulatory functions on gene expression. They negatively affect the expression of target genes by promoting either RNA degradation or translational inhibition. In recent years, converging studies have identified miRNAs as key regulators of oligodendrocyte (OL) functions. OLs are the cells responsible for the formation and maintenance of myelin in the central nervous system (CNS) and represent a principal target of the autoimmune injury in multiple sclerosis (MS).

**Methods:**

MiRAP is a novel cell-specific miRNA affinity-purification technique which relies on genetically tagging Argonaut 2 (AGO2), an enzyme involved in miRNA processing. Here, we exploited miRAP potentiality to characterize OL-specific miRNA dynamics in the MS model experimental autoimmune encephalomyelitis (EAE).

**Results:**

We show that 20 miRNAs are differentially regulated in OLs upon transition from pre-symptomatic EAE stages to disease peak. Subsequent in vitro differentiation experiments demonstrated that a sub-group of them affects the OL maturation process, mediating either protective or detrimental signals. Lastly, transcriptome profiling highlighted the endocytosis, ferroptosis, and FoxO cascades as the pathways associated with miRNAs mediating or inhibiting OL maturation.

**Conclusions:**

Altogether, our work supports a dual role for miRNAs in autoimmune demyelination. In particular, the enrichment in miRNAs mediating pro-myelinating signals suggests an active involvement of these non-coding RNAs in the homeostatic response toward neuroinflammatory injury.

## Background

MicroRNAs (miRNAs) belong to a class of ~ 23 nt small non-coding RNAs acting as important regulators of gene expression, both in plants and animals. They promote the post-transcriptional downregulation of target genes by pairing specific seed sequences located prevalently in the 3′UTRs of messenger RNAs and causing either RNA degradation or translational inhibition [1]. Over 70% of the mammalian transcriptome has been estimated to be under the control of miRNAs and they are involved in multiple different biological processes ranging from cell differentiation to apoptosis [2]. A pivotal role for miRNAs in the development and activity of the central nervous system (CNS) has been described, including neurogenesis, neurite outgrowth, and myelin maintenance [3].

Oligodendrocytes (OLs) are the glial cells deputed to the production of myelin sheaths in the CNS [4]. They originate from oligodendrocyte precursor cells (OPCs), following a maturation process that is tightly regulated both in space and time by specific signaling pathways and genetic programs [5]. Recent studies have demonstrated that epigenetic factors including miRNAs are key regulators of OPC differentiation [6, 7]. Thus, dysregulation of specific miRNAs in OLs has been suggested to coincide with the initiation and expression of several demyelinating disorders such as multiple sclerosis (MS).

MS is a chronic autoimmune disease of the CNS characterized by focal lymphocytic infiltration, myelin breakdown, gliosis, and axonal degeneration [8]. Numerous attempts have been carried out to elucidate the role of miRNAs in OL loss, mostly based on assessing their levels in demyelinated lesion specimens [9, 10]. However, the extreme complexity of CNS cyto-architecture prevented the univocal attribution of differentially regulated miRNAs to specific cell lineages. Indeed, most of the lesion-specific detected miRNAs are believed to belong to astrocytes, reflecting their overall abundance in MS lesions. In this study, we adopted the novel cell-specific miRNA purification technique miRAP [11] to comprehensively investigate OL-specific miRNA dynamics in the experimental autoimmune encephalomyelitis (EAE) paradigm, a disease model that recapitulates several features of MS pathology including demyelination dynamics [12, 13]. This technique relies on expressing an epitope-tagged version of Argonaute 2 (AGO2) in selected cell populations by means of the Cre/lox binary system. AGO2 physiologically binds miRNAs as part of the RNA-induced silencing complex (RISC), which mediates their interaction with the mRNA targets [14]. Thus, AGO2 precipitation allows the selective isolation of miRNAs from a single cell population within a complex tissue with high specificity.

Here, we show for the first time that a restricted group of 20 miRNAs undergoes differential expression in OLs upon transition from pre-symptomatic EAE stages to clinically manifested disease. Furthermore, by manipulating their levels in vitro, we found that six of them are able to modulate OPC differentiation via specific cellular pathways. Altogether, our work provides compelling evidence for a mechanistic role of miRNA dysregulation in autoimmune demyelination.

## Materials and methods

### Mouse strains

*Olig1-Cre* mice (B6;129S4-*Olig1*^*tm1*(*cre*)*Rth*^/J), tAGO2 mice (B6(Cg)-*Gt*(*ROSA*)*26Sor*^*tm1*(*CAG*-*GFP*/*Eif2c2*)*Zjh*^/J) and C57BL/6J mice were purchased from The Jackson Laboratory. The generation and characterization of tAGO2 and *Olig1*-Cre lines have been previously described [11, 15]. All mice were maintained on a pure C57BL/6J background and only females of 8–10 weeks of age were used for experiments. Mice were housed in a specific pathogen free (SPF) facility. All animal procedures were performed in compliance with experimental guidelines approved by the UCSF Committee on Animal Research (CAR).

### Cell lines

The Oli-neu cell line was a kind gift of Dr. Isobel Scarisbrick (Mayo Clinic, MN, USA) and Dr. Patrizia Casaccia (Icahn School of Medicine at Mount Sinai, NY, USA). Cells were grown on poly-d-lysine coated dishes (Corning) and maintained in 1:1 DMEM:Neurobasal medium (GIBCO/Invitrogen) supplemented with N-2 (GIBCO/Invitrogen), B-27 (GIBCO/Invitrogen), 2 mM glutamine, 1 mM sodium pyruvate, 20 mM HEPES, 25 μM 2-mercaptoethanol, and antibiotics (100 IU/mL penicillin and 100 mg/mL streptomycin) at 37 °C in a humidified atmosphere with 5% CO_2_.

To establish OPC cultures, we adapted a previously published protocol [16]. Briefly, brains were dissected from P3-P6 mice and cortex regions were isolated. The tissue was minced and digested with 5 mL papain solution at 37 °C for 20 min. The reaction was stopped with 1 mL of fetal bovine serum and the solution was pipetted a few times to create a cell suspension. The suspension was passed first through a 70 μm cell strainer and then through a 40 μm cell strainer (Corning) to remove cell clumps. OPC isolation was subsequently carried out with anti-O4 microbeads (Miltenyi), following the manufacturer’s instructions. OPCs were plated onto coverslips coated with poly-d-lysine and laminin, and they were maintained in SATO medium supplemented with 10 ng/mL human PDGF-AA (PeproTech) and 10 ng/mL human NT-3 (PeproTech). For OPC differentiation, PDGF-AA and NT-3 were replaced with 40 ng/mL human T3 (Sigma). In both conditions, OPCs were kept in an incubator at 37 °C in a humidified atmosphere with 5% CO_2_. The purity of the OPC cultures was confirmed by staining the cells 1 day after plating for glial fibrillary acidic protein (GFAP) and neuronal nuclei (NeuN), two markers for astrocytes and neurons respectively. Less than 5% GFAP positive cells and no NeuN positive cells were counted (Fig. S1).

Both Oli-neu and OPCs were transfected with the N-TER Nanoparticle siRNA Transfection System (Sigma), following the manufacturer’s instructions. Both miRNA mimics and mimic negative control (Dharmacon) were used at the final concentration of 10 nM. Transfection efficiency was between 60 and 70% as confirmed by fluorescence microscopy using an Alexa Fluor-conjugated miRNA mimic (Thermo).

### Antibodies

The following antibodies were used in this study: anti-AGO2 monoclonal antibody (H00027161-M01, Abnova); anti-Myc monoclonal antibody (sc-40, Santa Cruz); normal mouse IgG (sc-2025, Santa Cruz); anti-GFP monoclonal antibody (A-11120, Invitrogen); anti-GFP polyclonal antibody (600-401-215, Rockland Immunochemicals); anti-OLIG1 monoclonal antibody (75-180, NeuroMab); anti-PDGFRα monoclonal antibody (3174, Cell Signaling); anti-CNPase monoclonal antibody (5664, Cell Signaling); anti-GFAP monoclonal antibody (3670, Cell Signaling); anti-NeuN monoclonal antibody (ab177487, Abcam); anti-MBP monoclonal antibody (808403, BioLegend); anti-mouse IgG, HRP-linked antibody (7076, Cell Signaling); anti-rabbit IgG F(ab')2 Fragment Alexa Fluor 555 conjugate (4413, Cell Signaling); and anti-mouse IgG F(ab')2 Fragment Alexa Fluor 488 conjugate (4408, Cell Signaling).

### EAE induction

Active EAE was induced following previously published procedures [17, 18]. Briefly, 8–10-week-old female mice were injected subcutaneously with 100 μg of murine myelin oligodendrocyte glycoprotein 35-55 (MOG_35-55_) (EZBiolab), in complete Freund’s adjuvant (CFA) with 4 mg/mL *Mycobacterium tuberculosis* (DIFCO Laboratories). Mice also received 400 ng of pertussis toxin (LIST Biological Laboratories) intraperitoneally at the time of immunization and 48 h later. Control mice were mock injected with everything except the MOG peptide. For all experiments, animals were observed daily, and clinical signs were assessed as follows: 0, no signs; 1, decreased tail tone; 2, mild monoparesis or paraparesis; 3, severe paraparesis; 4, paraplegia; 5, quadriparesis; and 6, moribund or death.

### Histopathology

Mice were perfused with 4% paraformaldehyde (PFA) in phosphate-buffered saline (PBS) and spinal cords were dissected. Subsequently, tissues were post-fixed in 4% PFA for additional 48 h before processing for paraffin embedding. Transversal 5 μm sections were then cut and stained with hematoxylin and eosin (H&E) or luxol fast blue (LFB) in order to visualize cell infiltration and demyelination, respectively. Both tissue processing and staining were outsourced at the UCSF Mouse Pathology Core.

### Immunohistochemistry

Mice were perfused with 4% PFA in PBS before collecting the CNS. The tissues were post-fixed in 4% PFA for additional 4 h before being included in OCT for sectioning. Thick sections (40 μm) were cut with an OTF5000 cryostat (Hacker Instruments) and stained following a free-floating protocol. Briefly, the sections were permeabilized in 0.3% Triton XT-100 in PBS for 10 min at room temperature (RT), before being blocked in 10% normal goat serum (NGS) in PBS with 0.3% Triton for 1 h at RT. Sections were then incubated with a cocktail of primary antibodies anti-GFP (1:800) and anti-OLIG1 (1:300) in blocking buffer at 4 °C overnight (ON). The day after, the sections were washed three times with PBS supplemented with 0.05% Tween-20 (PBS-T) and incubated with Alexa Fluor-conjugated secondary antibodies (1:1000) in blocking buffer for 1 h at RT in the dark. After three final washes with PBS-T, the sections were mounted on glass slides with Vectashied with DAPI (Vector Laboratories). Co-localization studies were carried out at the UCSF Nikon Imaging Center, using a C1si spectral confocal microscopy equipped with EZ-C1 software (Nikon). Acquired images were processed using the ImageJ software (https://imagej.nih.gov).

### Immunocytochemistry

OPCs were seeded on 24 mm coverslips and transfected on the same day with the different miRNA mimics or negative control. The day after, the growth medium was replaced with differentiation medium and cells were cultured for additional 3 days before being fixed in 2% PFA in PBS for 10 min at RT. Cells were subsequently permeabilized and blocked in 10% NGS with 0.1% Triton XT-100 for 1 h at RT. After 3 washes in PBS, cells were incubated in primary antibodies (1:300) diluted in 1% bovine serum albumin (BSA) in PBS with 0.01% Triton XT-100 ON at 4 °C. The coverslips were washed three more times and incubated with Alexa Fluor-conjugated secondary antibody (1:500) in 1% BSA for 1 h at RT, in the dark. After three final washes, the coverslips were mounted on glass slides with Vectashied with DAPI (Vector Laboratories). Cells were imaged at the UCSF Nikon Imaging Center, using an Eclipse Ti-E microscope equipped with NIS-Elements software (Nikon). In each experiment, at least 3 fields per condition were imaged randomly and the total number of cells positive to the different markers was measured using the ImageJ software. At least 300 cells were counted in each experimental condition.

### Cell proliferation and toxicity assays

Oli-neu cells were seeded in poly-D-lysine coated 96-well plates (Corning) and transfected on the same day with the different miRNA mimics or negative control. After 72 h, cell proliferation was measured using the Cell Proliferation Kit II (Roche), incubating the cells with the XTT reagent for 4 h. Absorbance was finally read at 492 nm with a VersaMax plate reader (Molecular Devices). For cell toxicity measures, an aliquot of conditioned media (20 μL) was removed before adding the XTT reagent and the amount of adenylate kinase released by damaged cells was quantified with the ToxiLight Bioassay Kit (Lonza), according to the manufacturer’s instructions. Luminescent signals were read with a Gemini plate reader (Molecular Devices). Three independent experiments were carried out, running each miRNA mimic transfection in triplicate.

### Immunoprecipitation assays

Mouse CNS tissues (either brains or spinal cords) were homogenized in 10 volumes of cold lysis buffer (10 mM Tris-HCl pH 7.5; 150 mM NaCl; 1% NP-40) supplemented with complete protease inhibitors (Roche). After 10 min on ice, lysates were spun at 16,000×*g* and supernatants collected. Subsequently, tissue lysates were pre-cleared with 50 μL of protein G Dynabeads (Invitrogen) for 1 h at 4 °C, rotating. The lysates were then incubated with 50 μL of Dynabeads conjugated with 5 μg of anti-AGO2, anti-GFP, or anti-Myc antibody for 5 h at 4 °C, rotating. Negative controls were instead incubated with beads conjugated with the same amount of normal IgG. After the incubation, beads were washed five times with 500 μL of lysis buffer and then proteins were eluted from the beads by boiling each sample in Laemmli loading buffer for 5 min.

### Western blotting assays

Protein samples were separated onto by SDS-PAGE on 10% polyacrylamide gels and then blotted onto nitrocellulose membranes (Immobilion) at 100 V for 30 min. Membranes were subsequently blocked with 5% milk in Tris-buffered saline with 0.05% Tween-20 (TBS-T) for 1 h at RT. After blocking, membranes were incubated with primary antibodies diluted (1:1000) in blocking solution ON at 4 °C. The day after, the membranes were washed 3 times with TBS-T and incubated with horseradish peroxidase (HRP)-conjugated secondary antibodies (1:5000) in blocking solution for 1 h at RT. After extensive washing, membranes were incubated with Supersignal West Dura reagent (Thermo Scientific) and the chemiluminescent signals were detected using a Molecular Imager ChemiDoc XRS System equipped with Quantity One software (Bio-Rad).

### miRAP isolation

To isolate OL-specific miRNAs from the mouse spinal cord, we employed the miRAP technique as previously described [11]. Briefly, spinal cord tissues (pools of 5 mice) were homogenized in 10 volumes of cold lysis buffer (10 mM HEPES pH7.4, 100 mM KCl, 5 mM MgCl_2_, 0.5% NP-40, 1 m M DTT, 100 U/mL RNasin) supplemented with complete protease inhibitors (Roche). Lysates were spun at 13,000×*g* for 30 min at 4 °C, and the supernatants were incubated with 50 μL of protein G Dynabeads (Invitrogen) conjugated with an anti-Myc antibody for 5 h at 4 °C, rotating. Beads were then washed twice with low stringency NT2 buffer (50 mM Tris-HCl pH 7.5, 150 mM NaCl, 1 mM MgCl_2_, 0.5% NP-40, 1 mM DTT, 100 U/mL RNasin), and twice with high stringency NT2 buffer (50 mM Tris-HCl pH 7.5, 600 mM NaCl, 1 mM MgCl_2_, 0.5% NP-40, 1 mM DTT, 100 U/mL RNasin). Ribonucleic complexes on the beads were digested with 0.6 mg/mL proteinase K (Ambion) for 20 min at 55 °C. Finally, RNA was purified by acid phenol-cloroform (Ambion) extraction, followed by ON precipitation at − 80 °C in the presence of sodium acetate and GlycoBlue (Ambion). Pelleted RNA was washed once with 75% ethanol and resuspended in water for downstream processing.

### Small RNA sequencing

Next-generation sequencing libraries were constructed from miRAP samples using the QIAseq miRNA Library Kit (QIAGEN). The cel-miRNA-39 spike-in control (Norgen) was added to each sample. Briefly, miRNAs were ligated to 3′ and 5′ adapters, reverse-transcribed, and PCR-amplified using Illumina next-generation sequencing primers. The resulting libraries were finally sequenced on a HiSeq4000 platform (Illumina), generating at least 3 million 50-bp single-end reads per sample. Small RNA sequencing was outsourced at the UCSF Center for Advanced Technology. Data analysis was carried out following a validated pipeline. Initial QC involved the trimming of adaptors and filtering of low quality sequences and spike-in controls. The remaining sequences were then mapped to the mouse reference genome (mm10) using the BWA software, allowing no mismatch. The different miRNAs were annotated according to miRbase (version 22). To compare miRNA expression across datasets, the edgeR package was used [19]. Briefly, the “calcNormFactors” function was applied to calculate normalization factors to scale each library, and the “exact test” function was employed to perform pairwise differential expression between experimental conditions. Differences in miRNA expression were considered significant for fold changes ≥ 2 and false discovery rates (FDR) ≤ 0.05.

### RNA sequencing

Total RNA was isolated using the Quick-RNA Microprep Kit (Zymo Research). The poly(A) selected paired-end sequencing libraries were constructed using the mRNA Hyper Prep Kit (KAPA), following the manufacturer’s instructions. Next-generation sequencing was performed using a HiSeq X-ten platform (Illumina), generating at least 20 million 150-bp pair-end reads per sample. RNA sequencing was outsourced at Novogene Corporation, Inc (Sacramento, CA, USA). For the analysis, low-quality and Illumina adapter sequences were first trimmed using the Trimmomatic tool. Sequencing reads were then aligned to the mouse reference sequence (mm10) using hisat2-2.1.0. The htseq-count tool and Ensembl gtf file were used to count aligned reads for each gene. Differentially expressed genes between groups were identified using the edgeR package [19].

### Quantitative RT-PCR assays

Total RNA (including miRNAs) was isolated with the miRNeasy Mini Kit (QIAGEN). For mature miRNA quantification, 300 ng of RNA were reverse transcribed using the miScript II RT Kit (QIAGEN) and 3–5 ng of cDNA were used for each quantitative real-time PCR (qRT-PCR) reaction. Amplification of the different miRNAs was performed using target-specific miScript Primer Assays (forward primers) and the miScript SYBR Green PCR Kit (QIAGEN), which contains the miScript universal Primer (reverse primer) and QuantiTect SYBR Green PCR Master Mix. All qRT-PCR reactions were run in triplicate, and relative changes in miRNA expression between experimental conditions were determined according to the 2^−ΔΔCT^ method, using U6 snRNA levels as internal control for normalization. For the quantification of miR-124 and miR-219, the TaqMan MicroRNA Reverse Transcription Kit (Invitrogen) was used in combination with the MicroRNA Assay mmu-miR-124a and the MicroRNA Assay hsa-miR-219 (Invitrogen), and the TaqMan Universal Master Mix II (Invitrogen). All amplifications were performed on a Mx3005P thermocycler (Stratagene) or a 7900HT thermocycler (ABI).

For mRNA quantification, cDNA was synthesized using the SuperScript III First-Strand Synthesis System (Invitrogen), and qRT-PCR reactions were performed using the SYBR Green Real-Time PCR Master Mix (Invitrogen). Gene expression levels were analyzed using validated primers from the PrimerBank [20] database (Table S1). The 2^−ΔΔCT^ method was used to calculate mRNA levels relative to *Gapdh* expression.

### Bioinformatics analyses

Pathway analysis was carried out using Metascape, a web-based portal providing comprehensive gene annotation and enrichment analysis including GO processes, KEGG pathways, and the Reactome gene set (https://metascape.org) [21]. Considering that only downregulated genes from RNA-seq differential expression analysis were used as seeds, a nominal *P* value of 0.05 was used as cut-off [22].

The enrichment score in MS risk genes for each miRNA was calculated by the following formula: log2 (number of predicted target MS risk genes/number of expected genes). The number of expected genes was computed as: (number of total MS risk genes/number of total genes in TargetScanHuman v7.2 database) × number of miRNA predicted targets from TargetScanHuman v7.2 database [23]. In addition, we tested whether the calculated enrichment scores were greater than expected by chance as follows: we randomly sampled 11 miRNAs from the TargetScanHuman v7.2 database and calculated the permuted enrichment score; after repeating the randomization 1000 times, we computed the permutation *P* value for the true enrichment score as the number of permutations in which the permuted enrichment score was higher than the true enrichment score. Enrichment scores higher than 1 and *P* values equal or less than 0.05 were considered significant.

### Statistical analyses

Differences between means of two groups were assessed with two-tailed Student’s *t* test. Differences in the ratios between OPCs positive and negative to specific markers were assessed by Fisher’s exact test. *P* values equal to 0.05 or less were considered significant. Data were expressed as mean ± SEM.

## Results

### Generation of a conditional mouse line expressing tagged-AGO2 in OLs

**Fig. 1:**
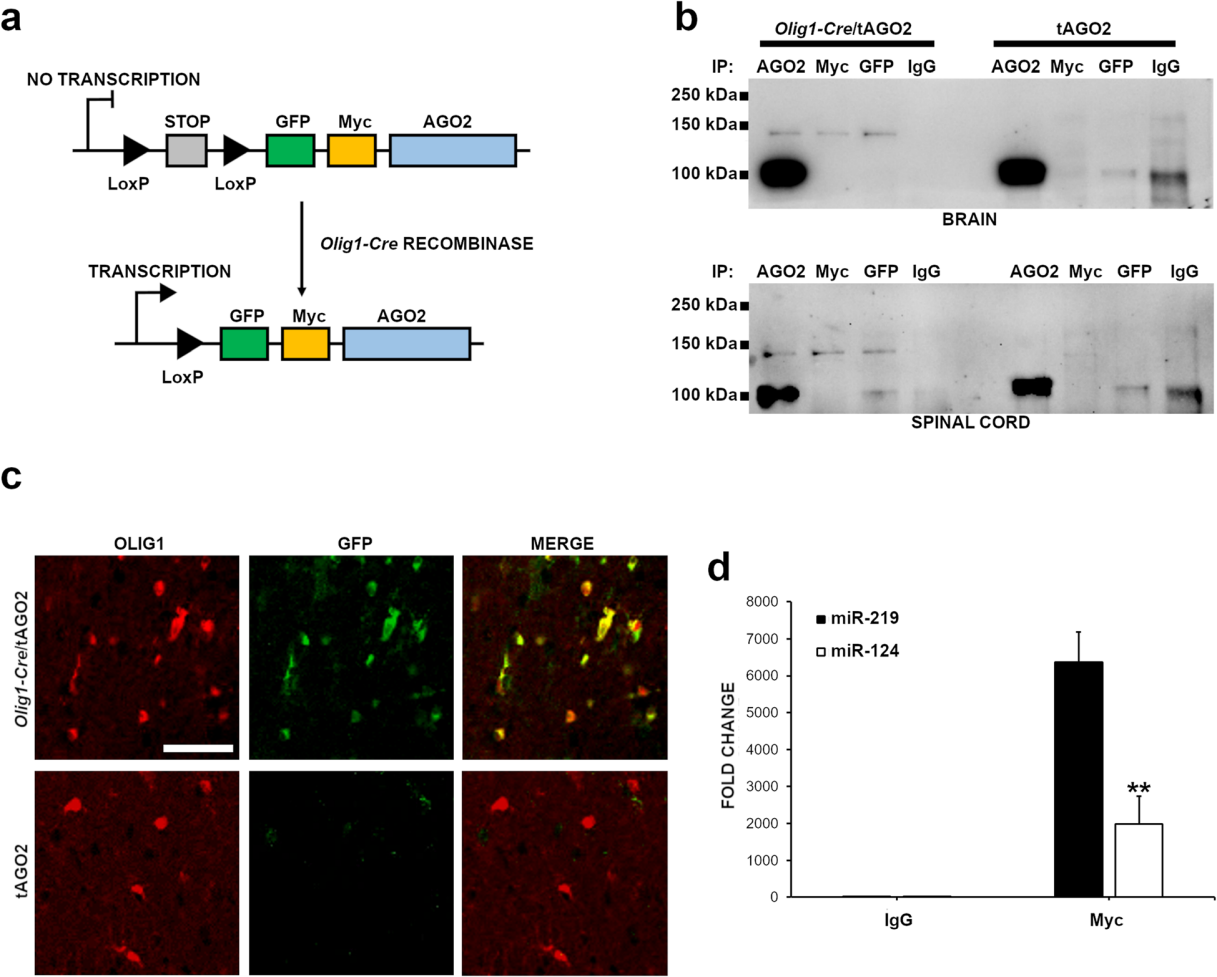
Conditional tAGO2 mice express epitope-tagged Argonaute 2 in OLs. **a** Cre-Lox system adopted to generate a conditional tAGO2 line specific for OLs. **b** Brain and spinal cord lysates from conditional tAGO2 and floxed AGO2 mice probed by immunoprecipitation (IP) with antibodies specific for AGO2, Myc and GFP. Normal IgGs were used as negative controls in mock IPs. All IP samples were analyzed by Western blotting using an antibody for AGO2. A signal corresponding to the epitope-tagged form of AGO2 was observed at around 120–130 kDa only in conditional tAGO2 mice. **c** Brain sections from both mouse lines were stained with an antibody for GFP (in green) and one for OLIG1 (in red) and inspected by confocal microscopy. Overlapping staining patters were observed exclusively in conditional tAGO2 mice while floxed controls were positive only to OLIG1 stain. Scale bar: 50 μm. **d** Brain samples from conditional tAGO2 mice were subjected to miRAP using antibodies for Myc and IgG as negative control. Purified samples were probed for miR-219 and miR-124 levels by qRT-PCR. As miR-219 is more enriched in OLs, its levels are higher in miRAP samples from Myc IPs in comparison to miR-124, which is more enriched in neurons. MiRNA levels are shown as fold differences (mean ± SEM) compared to IgG immunoprecipitated samples, coming from at least two independent miRAP experiments. **P ≤ 0.01

In order to perform miRAP isolation, we first generated a conditional mouse line expressing an epitope-tagged version of AGO2 exclusively in OLs by means of recombinase-activated gene expression (RAGE). We crossed the tAGO2 line carrying a stop codon-floxed *GFP*-*Myc*-*Ago2* transgene with a driver line in which the Cre recombinase is under the transcriptional control of *Olig1* promoter, a well-known pan-OL marker [24] (Fig. 1a). This line has been extensively employed for selective genetic manipulation in the OL lineage [15, 25–27]. To confirm the transgene expression in the conditional tAGO2 line (*Olig1*-*Cre*^*+*/−^, *GFP*-*Myc*-*Ago2*^+/−^), immunoprecipitation (IP) experiments were carried out on brain and spinal cord lysates from both conditional and floxed control mice, using antibodies specific for Myc, GFP, and AGO2. The IP samples were then probed by Western blot with an anti-AGO2 antibody (Fig. 1b). As expected, a signal corresponding to endogenous AGO2 (100 kDa) was observed in anti-AGO2 IP samples from both transgenic and control mice. Conversely, a signal compatible with tagged-AGO2 expected molecular weight (130 kDa) was detected only in the samples from transgenic mice, when probed with antibodies for AGO2, Myc, and GFP (Fig. 1b).

Subsequently, we analyzed the specificity of transgene expression within the CNS by confocal microscopy. Following a similar strategy to the original miRAP study [11], brain sections from both transgenic mice and controls were double stained with an antibody specific for OLIG1 and one for GFP (Fig. 1c). In transgenic mice, OLIG1 stain co-localized with the GFP signal, confirming that GFP-Myc-AGO2 is expressed exclusively in OLs. In contrast, no GFP^+^ cells were found in control mice (Fig. 1c).

Lastly, to confirm the robustness of the methodology, we assessed the levels of miR-124a and miR-219—the former being one of the most conserved and abundantly expressed miRNAs in the nervous system, particularly in neurons [28] while the latter is highly enriched in myelinating OLs [29]. Antibodies against Myc were employed for the OL-specific miRAP purification, whereas normal IgGs were used as negative controls. As expected, miR-219 levels in Myc immunoprecipitated samples were significantly higher than the relative miR-124 levels, confirming the enrichment of OLs specific miRNAs (Fig. 1d).

### EAE pathology in conditional tAGO2 mice

To confirm that conditional transgenic mice were susceptible to EAE, we immunized 8–10-week-old tAGO2 females with MOG peptide according to the protocol described in the “Materials and methods” section. Injected mice developed the first signs of EAE at 11 days post-immunization (dpi), reaching the peak of disease at 20 dpi and partially recovering at 25 dpi (Fig. S2a). The incidence of the disease was 100%. We also characterized the histopathological phenotype of EAE mice in terms of inflammation and demyelination. Hematoxylin and eosin (H&E) stain of lumbar sections of spinal cords from immunized mice at 30 dpi highlighted an extensive infiltration of lymphocytes in the parenchyma, while no cell foci were found in the spinal cord of control mice (Fig. S2b). Similarly, luxol fast blue (LFB) stain indicated extended myelin loss in the spinal cord of EAE mice while no signs of myelin breakdown were present in control mice (Fig. S2c).

### Specific miRNAs are dysregulated in OLs upon EAE

After confirming the susceptibility to EAE and classical phenotype in conditional tAGO2 mice, we performed a longitudinal miRAP experiment to describe the variation in OL-specific miRNA repertoire associated with disease progression. Spinal cord tissues (pools of 5 animals) were collected at baseline, pre-symptomatic stages (10 dpi), and disease peak (20 dpi). Mock injected controls were also collected at the same time points. MiRNAs were isolated from each sample and characterized by next-generation sequencing. We then calculated differences in miRNA expression between EAE and control samples at each time point (Table 1). In the cross-sectional analysis, no significant differences in miRNA levels were detected at 10 dpi. This is consistent with the notion that spinal cord pathology in the MOG_35-55_ EAE paradigm is time- and score-dependent, and negligible demyelination is detected before the clinical onset [30, 31]. Conversely, miR-142a-5p and miR-146a-5p were upregulated in EAE samples at 20 dpi, while miR-10b-5p levels were decreased.

**Table 1:** Differentially expressed miRNAs in cross-sectional comparisons

	log2(FC)	*P* value	FDR
EAE (10dpi) vs. Mock (10dpi)			
None	–	–	–
EAE (20dpi) vs. Mock (20dpi)			
mmu-miR-142a-5p	6.426	1.65E-05	0.0022
mmu-miR-146a-5p	3.278	0.00016	0.0076
mmu-miR-10b-5p	− 3.015	0.00016	0.0076

We also performed longitudinal comparisons to identify those miRNAs that are dynamically regulated along EAE progression (Table 2). In this second analysis, only miR-582-3p was found downregulated at 10 dpi as compared to baseline, in both EAE and control samples, thus suggesting a non-specific effect of EAE induction. Remarkably, 18 miRNAs were differentially regulated in OLs upon transition from pre-symptomatic to manifested EAE stages. Fifteen of them (miR-7654-3p, miR-146a-5p, miR-466i-3p, miR-6944-5p, miR-342-3p, miR-155-5p, miR-1193-5p, miR-221-3p, miR-203-3p, miR-1249-3p, miR-210-3p, miR-28a-3p, miR-667-3p, miR-3093-5p, and miR-8114) showed increased levels at 20 dpi as compared to 10 dpi. On the contrary, lower levels of miR-6983-5p, miR-219c-3p, and miR-190a-5p were measured at disease peak. It is worth mentioning that miR-342-3p, miR-221-3p, and miR-203-3p were differentially expressed with nominally significance also in the 20 dpi cross-sectional analysis. The remaining miRNAs were excluded from the cross-sectional comparison due to the strict statistical thresholds we established in our analytical pipeline. No miRNAs reached statistical significance in the comparison between 10 dpi and 20 dpi in mock-injected controls. Importantly, the expression of these 20 miRNAs follows distinct dynamics in whole spinal cord tissues without miRAP enrichment, further confirming the specificity of our approach (Table S2).

**Table 2:** Differentially expressed miRNAs in longitudinal comparisons

	log2(FC)	*P* value	FDR
EAE (10dpi) vs. BL			
mmu-miR-582-3p	− 3.02	0.00051	0.0455
Mock (10dpi) vs. BL			
mmu-miR-582-3p	− 3.034	8.36E-05	0.0191
EAE (20dpi) vs. EAE (10dpi)			
mmu-miR-7654-3p	4.91	1.67E-05	0.0057
mmu-miR-146a-5p	3.139	5.28E-05	0.0074
mmu-miR-466i-3p	3.44	7.09E-05	0.0074
mmu-miR-6944-5p	4.445	0.0001	0.0074
mmu-miR-342-3p	3.685	0.00013	0.0077
mmu-miR-155-5p	2.955	0.00028	0.0122
mmu-miR-1193-5p	3.857	0.00032	0.0122
mmu-miR-221-3p	2.31	0.00054	0.0168
mmu-miR-203-3p	2.268	0.00063	0.0182
mmu-miR-1249-3p	2.603	0.00082	0.0217
mmu-miR-210-3p	2.569	0.00092	0.0225
mmu-miR-28a-3p	2.405	0.00099	0.0225
mmu-miR-667-3p	2.598	0.00165	0.0354
mmu-miR-3093-5p	2.625	0.00236	0.0449
mmu-miR-8114	2.343	0.00236	0.0449
mmu-miR-6983-5p	− 4.023	9.10E-05	0.0074
mmu-miR-219c-3p	− 3.701	0.00051	0.0168
mmu-miR-190a-5p	− 2.563	0.00029	0.0122
Mock (20dpi) vs. Mock (10dpi)			
None	–	–	–

### The EAE-associated miRNAs are expressed in OLs in physiological conditions

**Fig. 2:**
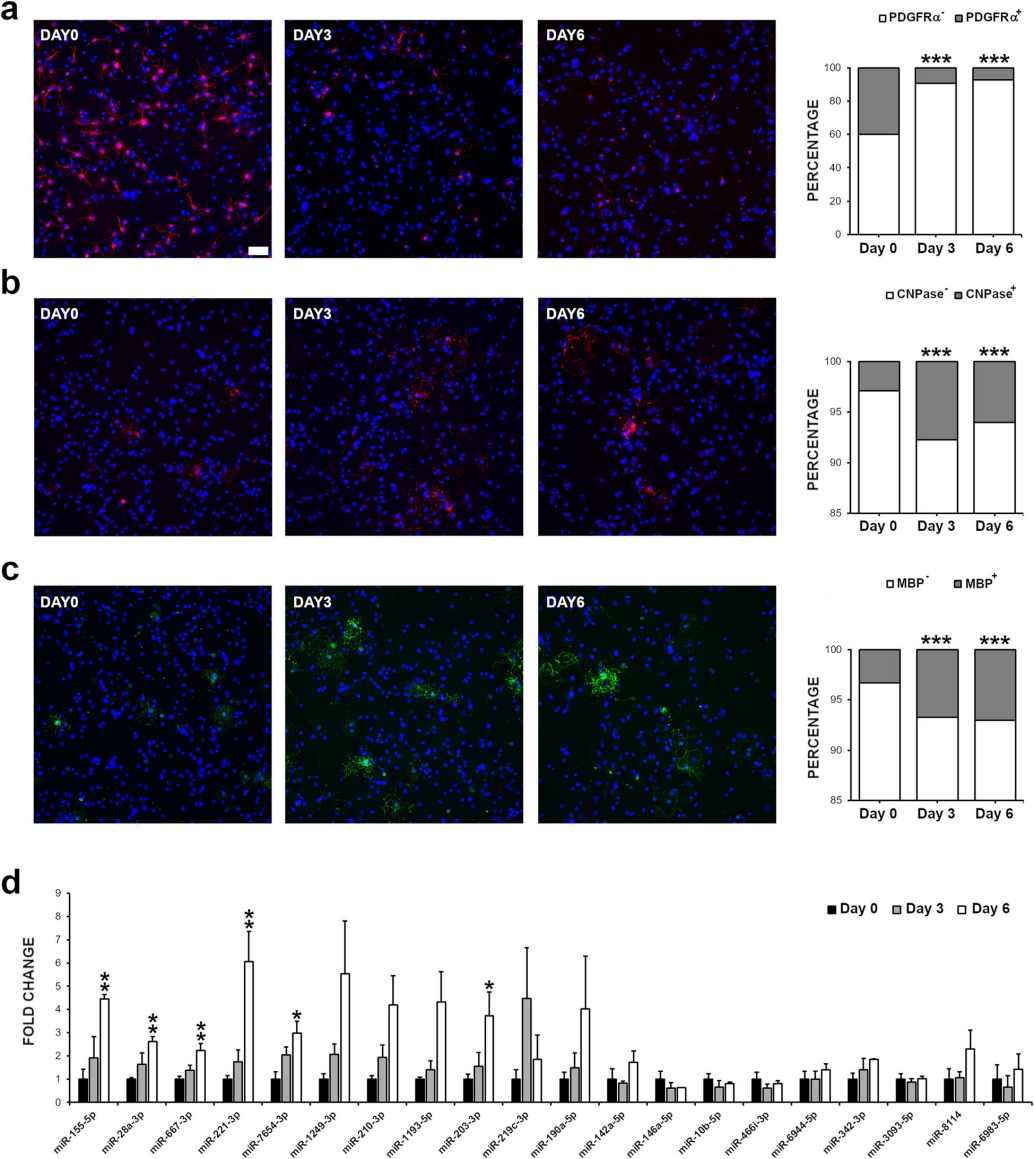
EAE-associated miRNAs are expressed in OLs. **a–c** The number of cells expressing different OL developmental markers was assessed by immunofluorescence in primary mouse OPC cultures. OPCs were fixed with PFA after 0, 3, and 6 days of in vitro differentiation and stained for the OPC marker PDGFRα (in red), the immature OL marker CNPase (in red), or the myelinating OL marker MBP (in green). Nuclei were counterstained with DAPI (in blue). The percentages of cells positive and negative to each marker are plotted. Statistical significance was evaluated by Fisher’s exact test on the total number of cells from two independent cultures. Scale bar: 50 μm. **d** Expression analysis by qRT-PCR of the 20 miRNAs identified by miRAP in OPCs incubated in differentiation medium for 0, 3, or 6 days. Expression levels are shown as fold differences (mean ± SEM) compared to day 0 (*n* = 3 cultures). *P ≤ 0.05, **P ≤ 0.01, ***P ≤ 0.001

Primary cultures of OPCs were established from postnatal mouse brains (P3-P6) and were differentiated in vitro by supplementing their growth media with triiodothyronine (T3). The acquisition of a mature OL phenotype was monitored by staining the cells in culture with markers for different developmental stages. Specifically, platelet-derived growth factor receptor α (PDGFRα) was used for OPCs, 2′,3′-cyclic nucleotide 3′ phosphodiesterase (CNPase) for immature OLs, and myelin basic protein (MBP) for myelinating OLs [32]. We detected 40.0% of PDGFRα-positive cells at day 0, and their number decreased to 9.3% and 7.1% after 3 and 6 days in differentiation medium (Fig. 2a). Conversely, 2.9% of cells were positive to CNPase at day 0, and their number increased to 7.8% and 6.0% after 3 and 6 days of culture (Fig. 2b). Similarly, 3.3% of cells were found positive for MBP at day 0, and their number increased to 6.7% at day 3 and 7.0% at 6 (Fig. 2c).

Upon verifying the robustness of the OL maturation model, we measured by qRT-PCR the abundance of all 20 EAE-associated miRNAs at the same time points of in vitro differentiation. As expected, all of them were expressed in OPCs. The levels of 6 miRNAs (miR-155-5p, miR-28a-3p, miR-667-3p, miR-221-3p, miR-7654-3p, and miR-203-3p) were significantly increased in cells at day 6 compared to day 0 (Fig. 2d). A trend for higher expression in mature OLs was also found for other 5 miRNAs (miR-1249-3p, miR-210-3p, miR-1193-5p, miR-219c-3p, and miR-190a-5p), although the differences did not reach statistical significance. The expression of the remaining 9 miRNAs did not change across the tested time points.

### Specific EAE-associated miRNAs control OPC differentiation

**Fig. 3:**
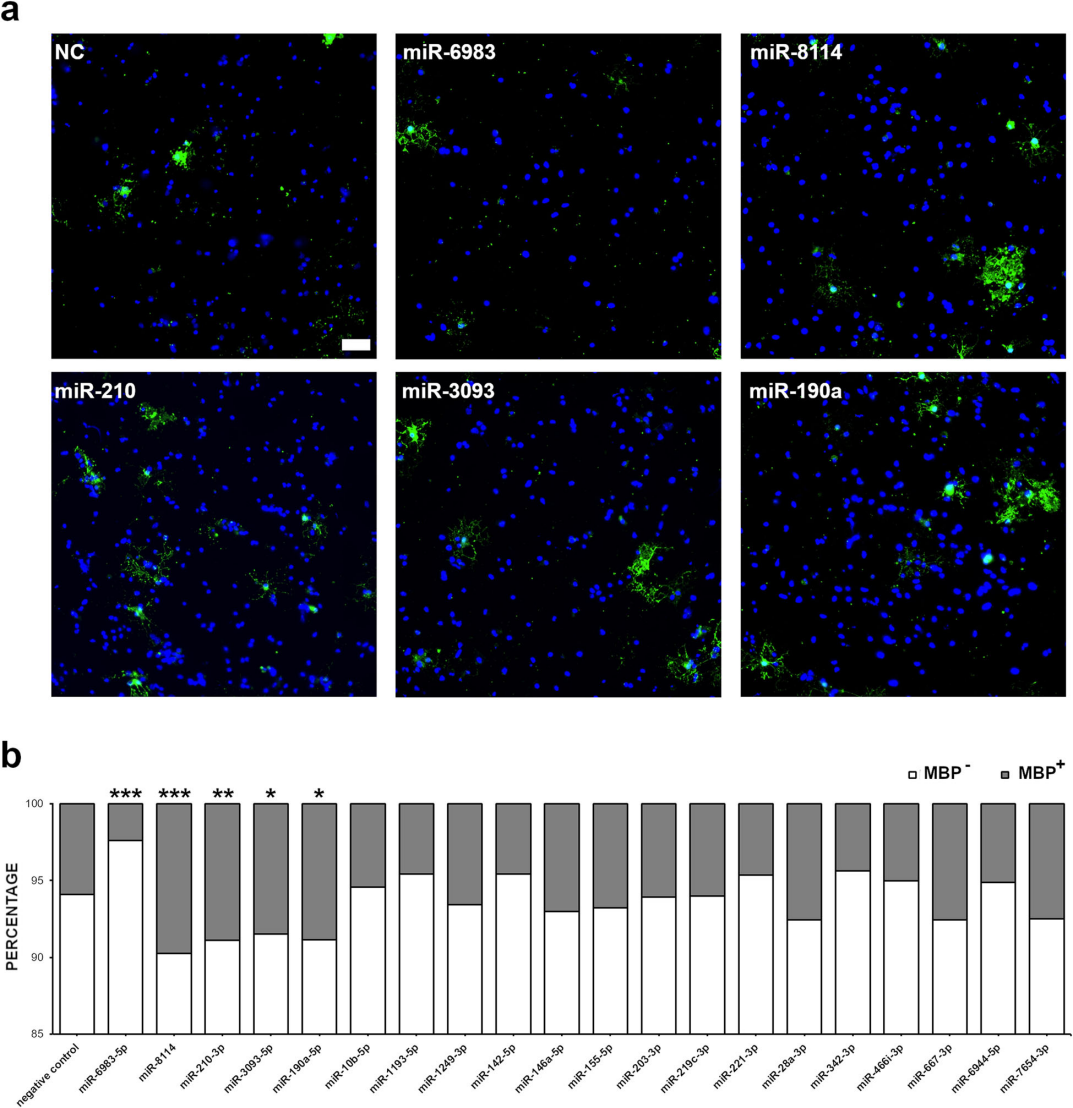
OPC maturation is controlled by specific EAE-associated miRNAs. **a** OPCs overexpressing mimics of the 20 EAE-associated miRNAs or a negative control (miR-67 from *C. elegans*) were differentiated for 3 days and the numbers of MBP^+^ OLs were assessed by immunofluorescence microscopy (green). Nuclei were counterstained with DAPI (blue). Representative images for the significant miRNAs are shown. Scale bar: 50 μm. **b** The percentages of MBP^+^ and MBP^-^ cells for each condition are plotted. Statistical significance was evaluated by Fisher’s exact test on the total number of cells from two independent transfections. *P ≤ 0.05, **P ≤ 0.01, ***P ≤ 0.001

Artificial mimics were next employed to efficiently overexpress the different miRNAs in primary OPCs. This was confirmed in preliminary experiments comparing a miR-142-5p mimic to a *Caenorhabditis elegans* miRNA control (data not shown, fold change: 475.87 ± 8.49). Transfected OPCs were subsequently incubated in differentiation medium for 3 days, before assessing the number of MPB^+^ cells by immunofluorescence microscopy. Immunocytochemistry analysis revealed that OPCs overexpressing miR-1193-5p, miR-190a-5p, miR-210-3p, miR-3093-5p, and miR-8114 possessed an increased propensity to differentiate as demonstrated by the higher percentages of MBP^+^ cells as compared to control (Fig. 3a, b). On the contrary, miR-6983-5p overexpression resulted in a significant decrease in the number of myelinating OLs (Fig, 3a, b). Since miR-1193-5p, miR-210-3p, miR-3093-5p, and miR-8114 are upregulated in OLs upon EAE, they presumably represent homeostatic responses to promote new myelin formation. Likewise, miR-6983-5p, which suppresses maturation when over expressed in vitro, is downregulated in vivo thus mediating another re-myelination signal. Conversely, miR-190a-5p is downregulated upon EAE and could be then associated to EAE pathogenesis via re-myelination inhibition.

To exclude these differences could be due to putative changes in the overall cell number, we assessed the effects of these 20 miRNAs on OPC proliferation and viability. For this purpose, we employed the immortalized murine Oli-neu cell line, a widely used model to study OPC biology [33, 34]. Oli-neu cells were transfected with the different miRNA mimics or with the negative control and let grow for 3 days before assessing their proliferation rates by means of XTT assay. No statistically significant differences were measured between cells expressing any of the 20 miRNA mimics as compared to control cells (Fig. S3a). In addition, no increase in cytotoxicity was detected upon miRNA mimic overexpression (Fig. S3b).

### EAE-associated miRNAs control distinct pathways involved in OL differentiation

**Fig. 4:**
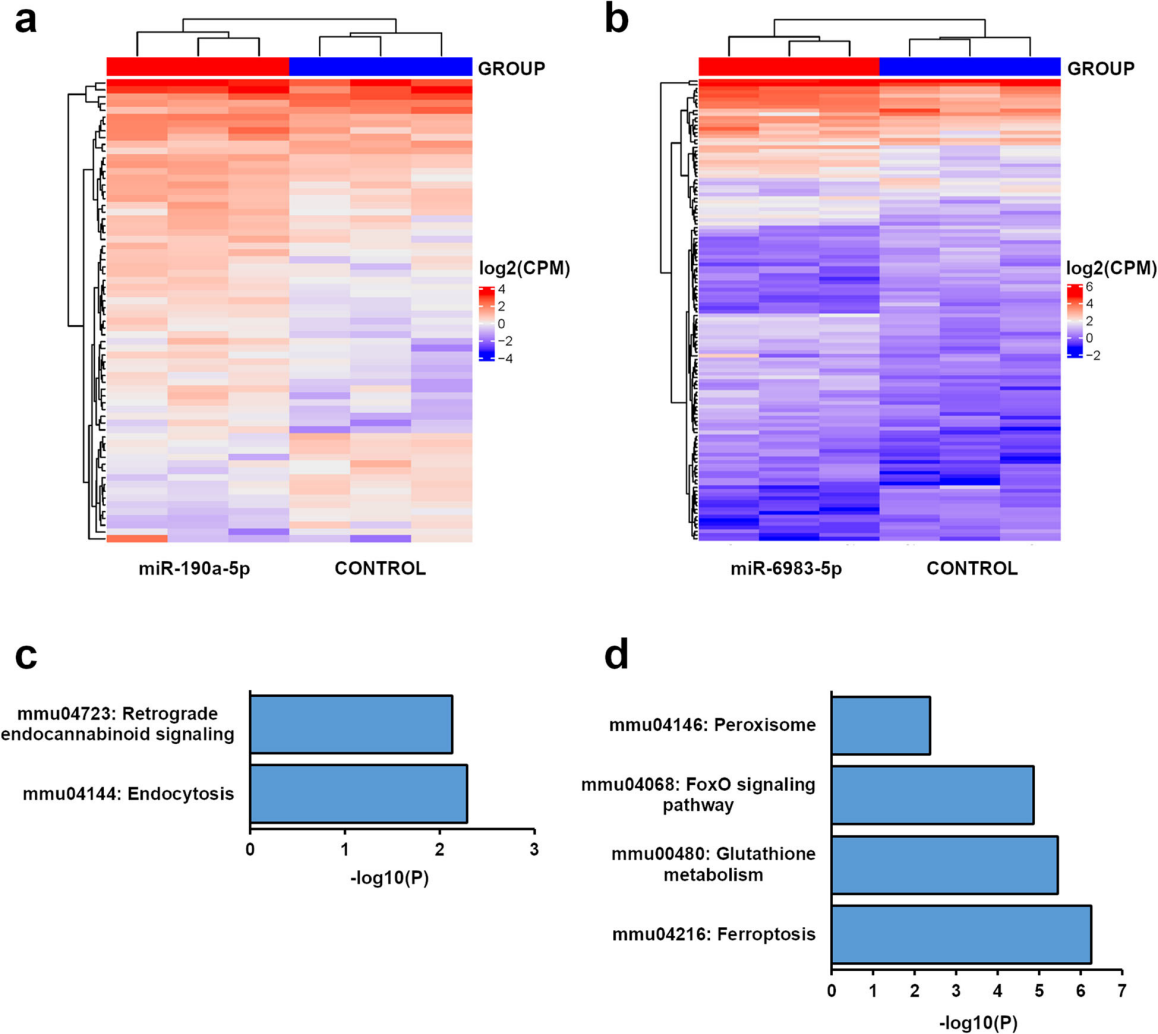
The effects of EAE-associated miRNAs are mediated by specific pathways. **a, b** Unsupervised hierarchical clustering of differentially expressed genes (DEGs, fold change ± 1.5) in differentiated OPCs overexpressing miR-190a-5p or miR-6983-5p as compared to control cells transfected with miR-67 mimic. The distances between samples were calculated using the Euclidean distance method (*n* = 3 transfections). **c**, **d** Histograms showing the most significant KEGG pathways for the DEGs associated to miR-190a-5p or miR-6983-5p overexpression. In this analysis, only the nominally significant down-regulated genes were used (*P* < 0.05), in order to isolate the core functions controlled by each miRNA in differentiated OPCs

A single miRNA can control up to hundreds of target genes [35]. Thus, we were keen to characterize the specific genetic networks and molecular pathways regulated by the EAE-associated miRNAs. In particular, we decided to analyze the putative pathogenic and protective effects mediated respectively by miR-190a-5p and miR-6983-5p, which represent the most significant differentially expressed miRNAs in each category. RNA-seq technology is a reliable approach to capture the genes subjected to the repressing activity of a specific miRNA [36]. Hence, detailed transcriptomic profiling of OPCs over-expressing the two miRNA mimics or the miR-67 control was carried out. Comparative analysis between miR-190a-5p and control identified 341 differentially expressed genes (DEGs) (235 upregulated and 106 downregulated), while 552 DEGs were found upon miR-6983-5p over-expression (323 upregulated and 129 downregulated). Differences in gene expression at the ± 1.5-fold-change threshold are sufficient to clearly segregate the different transcriptomic profiles by unsupervised hierarchical clustering (Fig. 4a, b).

To highlight the core of cellular functions directly affected by miRNA over-expression, we focused the subsequent bioinformatics analyses on the sub-groups of downregulated DEGs. KEGG pathway enrichment identified “endocytosis” as the most significant category associated to the 106 DEGs of miR-190a-5p (fold increase: 4.49, *P* = 5.14 × 10^−3^), mostly due to the downregulation of genes such as actin related protein 2/3 complex subunit 5 (*Arpc5*), EH domain containing 3 (*Ehd3*), and vesicle trafficking 1 (*Vta1*) (Fig. 4c). The term “ferroptosis” was instead the most enriched pathway for the 129 DEGs of miR-6983-5p (fold increase: 30.97, *P* = 5.58 × 10^−7^), as a consequence of the downregulation of ferritin light chain 1 (*Ftl1*), glutamate-cysteine ligase modifier subunit (*Gclm*), and heme oxygenase 1 (*Hmox1*) (Fig. 4d). Other significant pathways associated to this miRNA are “glutathione metabolism” (fold increase: 21.52, *P* = 3.56 × 10^−6^) due to the downregulation of glucose-6-phosphate 1-dehydrogenase (*G6pdx*) and glutamate-cysteine ligase modifier subunit (*Gclm*), and “FoxO signaling pathway” (fold increase: 11.54, *P* = 1.35 × 10^−5^) due to the downregulation of B cell lymphoma 6 (*Bcl6*) and catalase (*Cat*).

To explore the role of these miRNAs in vivo, we then tested the levels of the genes contributing to each GO category in EAE spinal cord at baseline, 10 and 20 dpi (Table S3). We found that *Arpc5*, *G6pdx*, *Hmox1*, *Prdx1*, *Ftl1*, and *Bcl6* are significantly upregulated in EAE mice at 20 dpi compared to mock injected controls. As both miR-190a-5p and miR-6983-5p are downregulated in OLs at the same EAE time point, these genes represent possible effectors of these two miRNAs in autoimmune demyelination.

### The EAE-associated miRNA targets are enriched in MS risk genes

Eleven miRNAs in the EAE-OL signature are conserved in the human genome (miR-10-5p, miR-1249-3p, miR-142-5p, miR-146-5p, miR-155-5p, miR-190-5p, miR-203a-5p, miR-210-3p, miR-221-3p, miR-28-3p, and miR-342-3p). We hence decided to assess possible overlaps with human disease. Specifically, we first computed all the predicted genetic targets for each of the 11 miRNAs, identifying 64 MS susceptibility genes [37] as potential miRNA targets (Table S4). An enrichment score was then calculated for each miRNA by comparing the observed results to those expected from random sampling, with two miRNAs showing statistically significant enrichments, miR-155-5p (score: 1.20, *P* = 0.05) and miR-210-3p (score: 1.68, *P* 0.01). As confirmed by cell-specific expression atlases [38, 39], 15/19 targets of miR-155-5p and 2/2 targets of miR-210-3p are expressed in OLs.

## Discussion

The molecules and signaling pathways associated with the OL degenerative process occurring upon autoimmune demyelination have been described in great detail [40]. Conversely, much less is known about the contribution of epigenetic mechanisms to OL survival and death. Here, we demonstrate that expression changes in the roster of OL-specific miRNAs correlate with the full manifestation of the disease in the EAE autoimmune demyelinating model. In vitro functional follow-up suggests that the miRNAs repertoire emerging following a neuroinflammatory challenge mediates both neuroprotective and detrimental signals for myelin integrity.

Previous attempts to characterize the miRNA repertoire in OLs relied primarily on laser-capture microdissection for cell isolation. However, this method of cell enrichment introduces significant mechanical stress on the specimens that might result in technical artifacts. Moreover, standard commercial kits for miRNA extraction are prone to generate numerous false positives due to mRNA fragmentation. In contrast, the miRAP technique adopted here allows the biochemical purification of highly enriched pools of physiologically active miRNAs from intact tissues, with minimal disruption. In addition, miRNAs from specific cellular domains can be isolated with this method, which is particularly important for cell types with complex morphologies and extended cytoplasmic processes such as OLs. MiRAP has been successfully employed to characterize the miRNA repertoires in a variety of CNS cell types including neurons, astrocytes, microglia, and infiltrating dendritic cells, both in health and disease [11, 41, 42]. The present study is the first exploiting this approach to profile miRNA dynamics in OLs along EAE progression.

Two miRNAs have been previously reported as implicated in OL functions in the context of MS CNS pathology. MiR-219 and miR-338 were found downregulated in MS lesions [9], and proven to function as key promoters of OL differentiation [7]. Specifically, miR-219 and miR-338 induce OL maturation by repressing the transcription factors *Sox6* and *Hes5*. MiR-219 also plays a central role in myelin maintenance through the regulation of the levels of the fatty acid elongase 7 (ELOVL7) enzyme [43]. In our dataset, miR-219c (a miRNA belonging to the same family of miR-219) was found downregulated at disease peak, suggesting a function comparable to miR-219. More recently, miR-23a, miR-27a, and miR-146a have been highlighted as additional regulators of OL differentiation [44–46]. It was also shown that miR-146a is upregulated along with miR-142 and miR-466b in OPCs upon hypoxia-ischemia (HI) conditions [47]. MiR-146a, miR-142, and the closely related miR-466i are similarly upregulated in our screening, suggesting possible overlaps in OL response to both insults. With the exception of these four miRNAs, the other 16 have not been reported before being associated with OPC maturation, and likely underlie EAE-specific events that do not take place upon physiological OL development. Notably, more than half of the miRNAs in the EAE signature have a human ortholog and two of them show a significant enrichment in MS risk gene targets that are expressed in OLs. This is particularly relevant in light of recent findings on OL intrinsic contribution to MS risk via disruption of key genes involved in transcription regulation [48]. However, the direct translation of animal model results to the human disease should be always cautious due to the substantial differences existing between the two entities—unlike MS, EAE does not develop spontaneously but it is artificially induced.

The in vitro differentiation experiments reported here identified six novel miRNAs affecting OL maturation. Surprisingly, we did not detect significant effects for miR-146a, which was shown to have a positive influence on OPC differentiation in similar assays [45]. Different culture conditions could account for such discrepancy. For example, the reported activity of miR-146a was measured after 5 days in culture, while in our setup we counted MBP^+^ cells after 3 days in culture in order to capture earlier and possibly stronger effects. MiR-1193 was previously shown to suppress proliferation and invasion of cancer cells through directly targeting transmembrane 9 superfamily member 3 (*TM9SF3*), and insulin-like growth factor 2 mRNA binding protein 2 (*IGF2BP2*) [49, 50]. MiR-210 has been associated with the progression of various types of cancer as well as with the regulation of energy metabolism [51, 52]. In addition, miR-210 regulates microglia-mediated neuroinflammation in hypoxic-ischemic encephalopathy [53].

To gain additional insights into the role of the newly identified EAE-associated miRNAs in the context of OL and myelin biology, we sequenced the transcriptome of OPCs over-expressing the mimics of miR-190a or miR-6983, miRNAs mediating respectively anti- and pro-differentiation signals, affording the identification of cellular pathways not previously connected to OPC maturation. The endocytosis process associated to miR-190a is important for OPC plasma membrane remodeling, while terminally differentiated OLs exhibit reduced endocytic activity [54]. Therefore, we propose that miR-190a downregulation upon EAE may halt the formation of new myelin as a result of endocytosis aberrant activation. On the other hand, ferroptosis is a non-apoptotic form of cell death that can be triggered by glutathione biosynthesis inhibition, and both represent the most significant pathways associated with miR-6983 [55]. Myelin is particularly enriched in iron and its aberrant deposition has been linked to neuroinflammatory damage in both EAE and MS [56]. Thus, miR-6983 downregulation upon EAE may constitute a feedback response to enhance OL survival via increased glutathione production and concomitant ferroptosis inhibition. Another cascade associated with miR-6983 is FoxO signaling. Remarkably, FoxO1 activation is required for OL regeneration after neonatal hypoxia [57]. Hence, the protective effects of miR-6983 downregulation may additionally work through the positive regulation of this pathway, further supporting the putative intersections between these two pathological conditions.

## Conclusions

In summary, our work is consistent with a scenario in which neuroinflammatory stress induces in OLs both homeostatic and degenerative responses. Both effects are mediated by a relatively small number of miRNAs, acting as high-raked regulators of underlying extended gene networks. Strategies aiming at manipulating the levels of protective miRNAs in the CNS of MS patients may pave the way to innovative therapies for enhancing the endogenous mechanisms of remyelination. This is in line with recent evidence showing alleviation of EAE severity upon over-expressing specific miRNAs [58].

A caveat in our study is the usage of an *Olig1*-Cre driver to generate the tAGO2 mice. Since *Olig1* is expressed along OPC development, from early precursors to myelinating OLs, our screening does not have the granularity to discriminate the EAE-associated miRNA dynamics at the different developmental OL stages. Future studies will increase the screen resolution by generating a panel of conditional tAGO2 lines employing Cre drivers specific for the different OL sub-populations.

## Supplementary information

**Additional file 1: Figure S1.** Purity of mouse OPC primary cultures. OPCs were fixed with PFA 24 hours after plating and stained for the astrocyte marker GFAP (in green) or the neuronal marker NeuN (in red). Nuclei were counterstained with DAPI (in blue). The percentages of cells positive and negative to each maker were plotted. Scale bar: 50 μm.

**Additional file 2: Figure S2.** EAE phenotype in conditional tAGO2 mice. (a) 8-10 weeks old conditional tAGO2 females were immunized with MOG_35-55_ peptide as detailed in the “Materials and methods” section and scored daily up to 30 days post-injection (dpi). Controls were mock immunized with everything but the peptide. Mean scores ± SEM are plotted (n=10 per group). (b-c) Histopathological analysis of representative spinal cords from EAE and control mice at 30 dpi using hematoxylin and eosin (H&E) or luxol fast blue (LFB). H&E stain highlights massive lymphocytic infiltration in the parenchyma of immunized mice. LFB stain (light blue) instead depicts extensive loss of myelin in the white matter. LFB sections were counterstained with Cresyl violet (dark blue). Scale bar: 200 μm.

**Additional file 3: Figure S3.** The EAE-associated miRNAs do not affect the proliferation and survival of the Oli-neu cell line. (a) The 20 miRNAs or the *C. elegans* negative control were overexpressed in Oli-neu cells and, after 72 hours, cell growth was assessed by XTT assay. No statistically significant differences in cell proliferation were measured in miRNA mimic-expressing cells compared to control. (b) An aliquot of conditioned media was tested at the same time point for the levels of adenylate kinase using the ToxiLight assay, as a proxy of cell damage. No differences were found between cells expressing the 20 miRNA mimics and the control. Results are plotted as fold changes to the control (mean FC ± SEM) and derive from three independent transfections.

**Additional file 4: Table S1.** List of primers used in qRT-PCR validation experiments.

**Additional file 5: Table S2.** Expression profiles of the 20 EAE-associated miRNAs in whole spinal cord tissues along EAE progression.

**Additional file 6: Table S3.** Expression profiles of miR-190a-5p and miR-6983-5p target genes in whole spinal cord tissues upon EAE (10 dpi and 20 dpi).

**Additional file 7: Table S4.** Predicted MS risk gene targets for the human orthologs of the EAE-associated miRNAs.

## Data Availability

The datasets used and/or analyzed during the current study are available from the corresponding author on reasonable request.

## Abbreviations

*Arpc5*: Actin-related protein 2/3 complex subunit 5
AGO2: Argonaute 2
*Bcl6*: B cell lymphoma 6
BSA: Bovine serum albumin
CAR: Committee on Animal Research
*Cat*: Catalase
CFA: Complete Freund’s adjuvant
CNPase: 2',3'-Cyclic nucleotide 3' phosphodiesterase
CNS: Central nervous system
DEGs: Differentially expressed genes
dpi: Day post-immunization
EAE: Experimental autoimmune encephalomyelitis
*Ehd3*: EH domain containing 3
ELOVL7: Fatty acid elongase 7
*Gclm*: Glutamate-cysteine ligase modifier subunit
GFAP: Glial fibrillary acidic protein
*Gclm*: Glutamate-cysteine ligase modifier subunit
*G6pdx*: Glucose-6-phosphate 1-dehydrogenase
H&E: Hematoxylin and eosin
HI: Hypoxia-ischemia
*Hmox1*: Heme oxygenase 1
*IGF2BP2*: Insulin-like growth factor 2 mRNA binding protein 2
IP: Immunoprecipitation
LFB: Luxol fast blue
miRNA: MicroRNA
MS: Multiple sclerosis
MBP: Myelin basic protein
MOG: Myelin oligodendrocyte glycoprotein
NeuN: Neuronal nuclei
NGS: Normal goat serum
OL: Oligodendrocyte
ON: Overnight
OPC: Oligodendrocyte precursor cell
PFA: Paraformaldehyde
PBS: Phosphate-buffered saline
PDGFRα: Platelet-derived growth factor receptor α
qRT-PCR: Quantitative real-time PCR
RAGE: Recombinase-activated gene expression
RISC: RNA-induced silencing complex
RT: Room temperature
*Slc40a1*: Solute carrier family 40 member 1
SPF: Specific pathogen free
T3: Triiodothyronine
*TM9SF3*: Transmembrane 9 superfamily member 3
*Vta1*: Vesicle trafficking 1
