# Supplementary figures and images for: Oligodendrocyte-specific Argonaute profiling identifies microRNAs associated with experimental autoimmune encephalomyelitis
Source: J Neuroinflammation. 2020 Oct 12;17:297. doi: 10.1186/s12974-020-01964-5 (PMC7552381; doi:10.1186/s12974-020-01964-5)

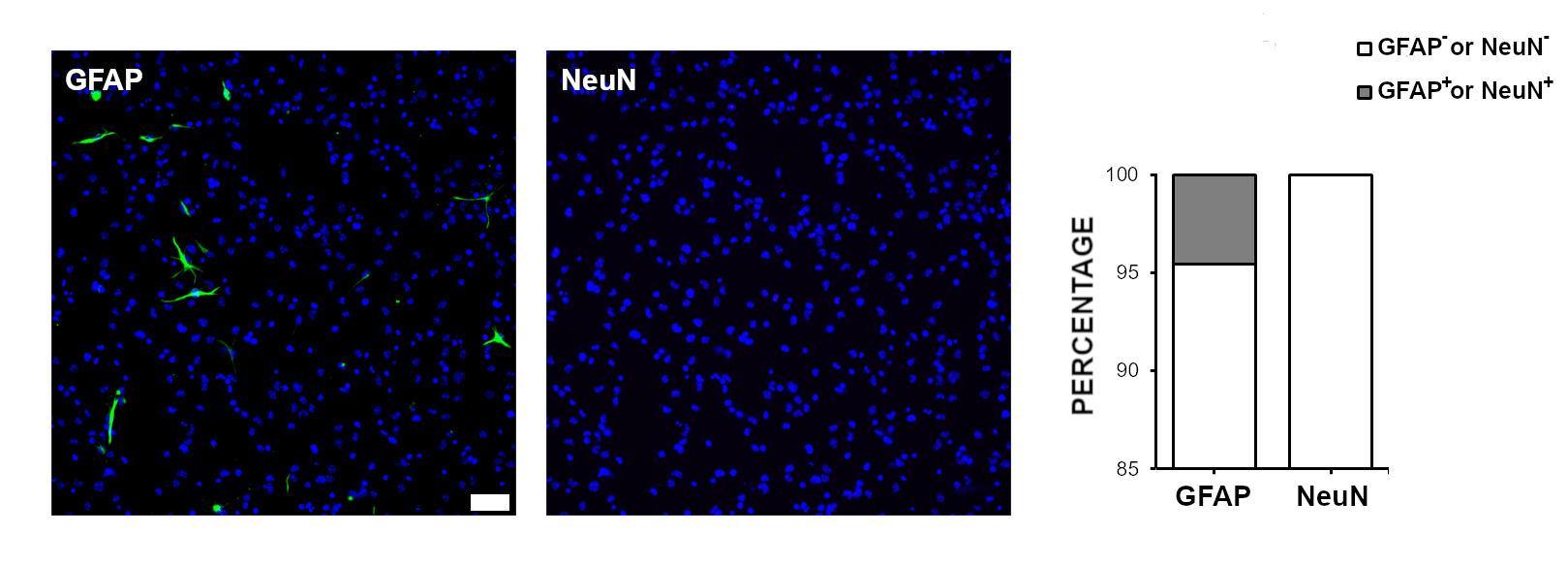

Supplement: Supplementary file 1 — Additional file 1: Figure S1. Purity of mouse OPC primary cultures. OPCs were fixed with PFA 24 hours after plating and stained for the astrocyte marker GFAP (in green) or the neuronal marker NeuN (in red). Nuclei were counterstained with DAPI (in blue). The percentages of cells positive and negative to each maker were plotted. Scale bar: 50 μm. [file 12974_2020_1964_MOESM1_ESM.tiff]

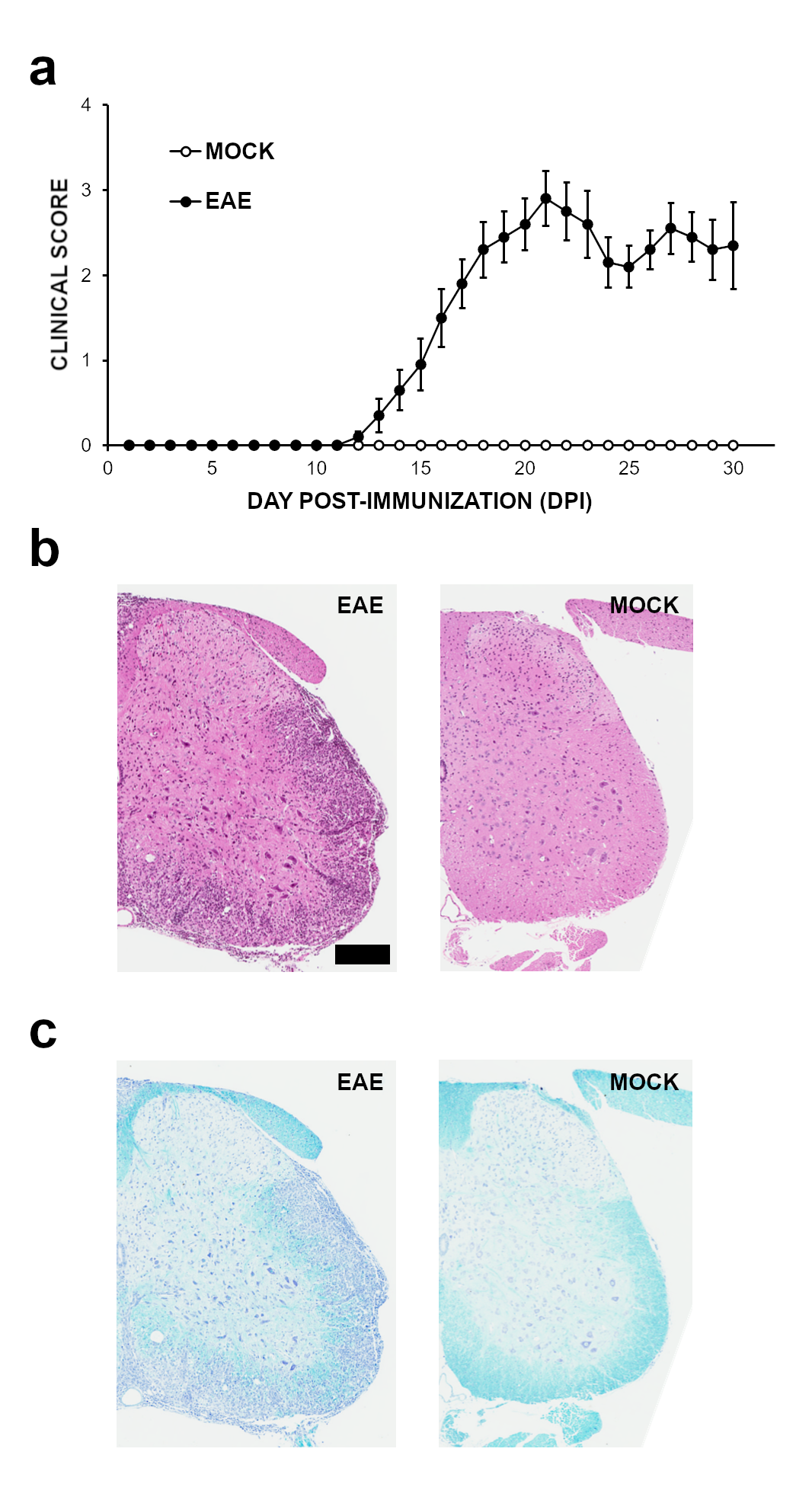

Supplement: Supplementary file 2 — Additional file 2: Figure S2. EAE phenotype in conditional tAGO2 mice. (a) 8-10 weeks old conditional tAGO2 females were immunized with MOG35-55 peptide as detailed in the “Materials and methods” section and scored daily up to 30 days post-injection (dpi). Controls were mock immunized with everything but the peptide. Mean scores ± SEM are plotted (n=10 per group). (b-c) Histopathological analysis of representative spinal cords from EAE and control mice at 30 dpi using hematoxylin and eosin (H&E) or luxol fast blue (LFB). H&E stain highlights massive lymphocytic infiltration in the parenchyma of immunized mice. LFB stain (light blue) instead depicts extensive loss of myelin in the white matter. LFB sections were counterstained with Cresyl violet (dark blue). Scale bar: 200 μm. [file 12974_2020_1964_MOESM2_ESM.tiff]

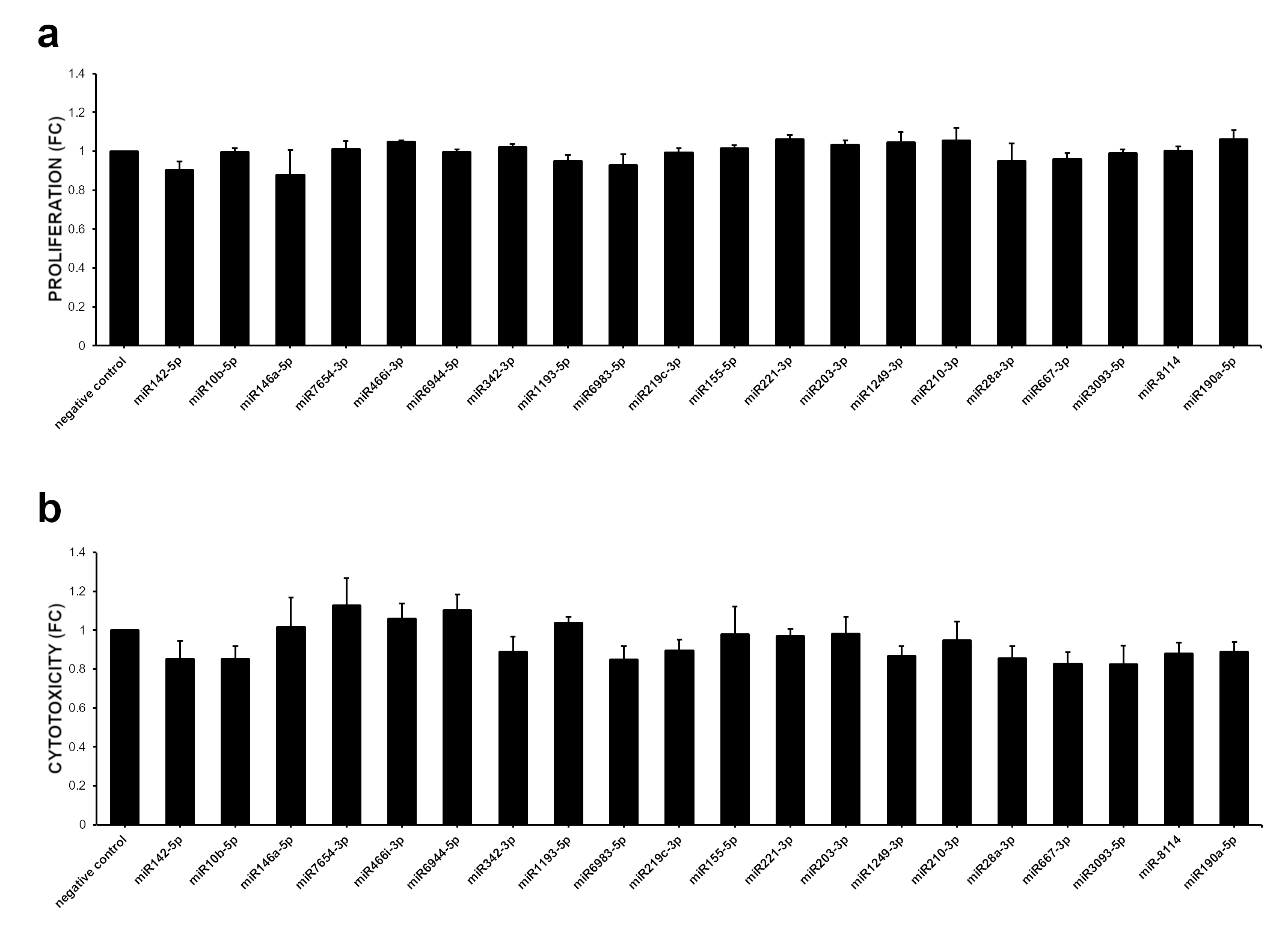

Supplement: Supplementary file 3 — Additional file 3: Figure S3. The EAE-associated miRNAs do not affect the proliferation and survival of the Oli-neu cell line. (a) The 20 miRNAs or the C. elegans negative control were overexpressed in Oli-neu cells and, after 72 hours, cell growth was assessed by XTT assay. No statistically significant differences in cell proliferation were measured in miRNA mimic-expressing cells compared to control. (b) An aliquot of conditioned media was tested at the same time point for the levels of adenylate kinase using the ToxiLight assay, as a proxy of cell damage. No differences were found between cells expressing the 20 miRNA mimics and the control. Results are plotted as fold changes to the control (mean FC ± SEM) and derive from three independent transfections. [file 12974_2020_1964_MOESM3_ESM.tiff]
